# Work-Related Stress and Psychological Distress among Law Enforcement Officers: The Carolina Blue Project

**DOI:** 10.3390/healthcare12060688

**Published:** 2024-03-19

**Authors:** Nayeon Lee, Ya-Ke Wu

**Affiliations:** 1 School of Nursing, University of North Carolina at Chapel Hill, Chapel Hill, NC 27599, USA; nlee@unc.edu; 2 Department of Psychiatry, University of North Carolina at Chapel Hill, Chapel Hill, NC 27599, USA

**Keywords:** police force, burnout, operational police stress, depression, anxiety, PTSD, North Carolina, rural county, toxic materials

## Abstract

Law enforcement is a stressful occupation that places significant psychological demands on those serving in this role. However, little is known about the severity of work-related stress and psychological distress among law enforcement officers (LEOs) in North Carolina (NC). This cross-sectional study examined the severity of work-related stress and psychological distress among 283 LEOs in NC. The Maslach Burnout Inventory, the Operational Police Stress Questionnaire, the Depression, Anxiety, and Stress Scale, and the Post-Traumatic Stress Disorder (PTSD) Checklist were used to assess burnout, operational police stress, depression, anxiety, stress, and PTSD among LEOs. Descriptive statistics, independent *t*-tests, Mann–Whitney U tests, one-way ANOVA, and Kruskal–Wallis tests were performed. Rural and male LEOs reported higher burnout levels related to depersonalization (i.e., increased mental distance from one’s job) compared with their urban and female counterparts. LEOs exposed to toxic materials or performing patrol duties exhibited higher operational police stress levels than those who did not. Caucasian LEOs exhibited higher depression, anxiety, and stress than their African American counterparts. Rural LEOs and LEOs who were exposed to toxic materials displayed higher levels of PTSD than their counterparts. Our findings highlight the need for increased mental health support and better working environments for LEOs.

## 1. Introduction

Law enforcement is an inherently stressful occupation that places significant emotional and psychological demands on those serving in this role [1,2,3,4]. Law enforcement officers (LEOs) are inevitably exposed to diverse traumatic and stressful situations, including engaging in shooting incidents, investigating crime scenes with deceased individuals, and dealing with traffic fatalities, child abuse/neglect, or exposure to illicit drugs [5,6,7]. Constant exposure to violence, trauma, and crisis situations could lead to work-related stress and psychological distress, such as burnout, chronic stress, depression, anxiety, post-traumatic stress disorder (PTSD), and other mental health disorders [8,9,10,11]. For example, Craddock and Telesco [6] found that up to 44% of the LEOs (total *n* = 408) in their study reported having nightmares, challenges in maintaining focus, and symptoms of agitation as a consequence of recurring traumatic memories during their duty. Another study on 434 LEOs in the ninth-largest police department in the United States (U.S.) found that 12% of participants had received a mental health diagnosis, such as anxiety, depression, and PTSD, while 26% reported experiencing present symptoms of mental illness in the preceding two weeks [12]. Addressing work-related stress and psychological distress among LEOs is imperative to preventing and ensuring the overall well-being of those tasked with upholding public safety and health.

Race plays a significant role in the experience of work-related stress and psychological distress among LEOs [13]. Research findings revealed that Caucasian officers were more likely to report higher levels of work-related stress than African American and other-race officers [14,15,16]. Similarly, He et al. [17] found that Caucasian male officers had elevated levels of somatization, anxiety, and depression compared with their African American male counterparts. However, LEOs’ sources of perceived work-related stress and psychological distress were found to differ depending on race. African American officers showed a greater tendency to have higher stress levels related to personal interaction with colleagues compared with their Caucasian counterparts, while Caucasian officers had higher stress levels due to departmental cultures than African American officers [13]. Furthermore, individuals of racial or ethnic minority backgrounds who serve as officers encounter distinct challenges associated with racial bias, discrimination, and community tensions [18,19]. Therefore, understanding the impact of race on work-related stress and psychological stress in LEOs could contribute to creating a more inclusive and equitable law enforcement system that fosters mental well-being among all officers, regardless of their racial or ethnic backgrounds.

The presence of geographical variations, specifically in urban and rural environments, could have distinct effects on the degrees of work-related stress and psychological distress experienced by LEOs [20,21,22]. While it is evident that both workplaces are characterized by persistent pressure and frequent occurrences, there are disparities in the prevalence of such incidents and the level of work-related stress and psychological distress experienced by LEOs. A report published by the National Criminal Justice Reference Service in 2017 indicated that urban LEOs encounter a higher frequency of incidents requiring investigation in cases of rape, sexual assault, robbery, and aggravated assault compared with their rural counterparts [23]. In an aligned study undertaken by Husain [21], the findings revealed that urban LEOs had higher levels of depression, anxiety, and stress than rural LEOs. However, the relative scarcity of mental health resources and training in rural areas may hinder access to support services, increasing their psychological distress [24]. If psychological distress remains unaddressed, it could have negative impacts on their intellectual capacity and job performance, such as reduced productivity, poor decision-making, and higher turnover [25,26].

Prior studies have suggested that exposure to toxic materials (e.g., cocaine, heroin, or fentanyl) on LEOs’ duties influences their work-related stress and psychological distress [27,28]. LEOs risk exposure to toxic materials in the form of solids, liquids, or gases in several ways, including inhalation hazards, direct-contact risks, ingestion of toxic materials, or ocular exposure risks [29]. As per the U.S. Federal Bureau of Investigation, sixty LEOs lost their lives in the line of duty from January to December 2023, with 16 of them sustaining injuries as a result of exposure to toxic materials [30]. The constant threat or awareness of exposure may contribute to heightened stress levels, as employees grapple with concerns about their health and well-being [31,32].

Although the effects of race, geographical variations, and experience with toxic materials on duty on LEOs’ work-related stress and psychological distress are present in the literature, to our knowledge, no studies have investigated them in conjunction with LEOs in North Carolina (NC). NC ranks 16th in the U.S. for crime rate [33]. The high rate of crime in NC may worsen the work-related stress and psychological distress experienced by law enforcement officers. Thus, the purpose of this cross-sectional study, The Carolina Blue Project, was to explore the level of work-related stress and psychological distress among LEOs in NC. The study investigated various factors such as race, geographical location, and exposure to toxic materials to determine the severity of burnout, operational police stress, depression, anxiety, stress, and PTSD symptoms experienced by LEOs. We also measured and included the comparisons of work-related stress and psychological distress based on sex, education, job category, and whether they served in leadership positions, were required to perform rotation shifts, or were required to perform patrol duties. Examining these variables helped identify potential disparities and unique stressors that may be associated with different socio-demographics and work-related variables [34,35].

## 2. Materials and Methods

### 2.1. Research Design and Setting

The Carolina Blue Project is a cross-sectional study investigating the severity of work-related stress and psychological distress among LEOs in NC in 2023. The project encompassed urban counties (i.e., an average population density exceeding 750 people per square mile), suburban counties (i.e., an average population density between 250 and 750 people per square mile), and rural counties (i.e., an average population density of 250 people per square mile or less) across the state [36]. There are 100 counties in NC (6 urban, 16 suburban, and 78 rural counties) and the total number of LEOs in NC was 37,040 in 2022 [37]. It is important to note that there are no data available to show the population proportions of LEOs by county in NC. However, the available data on the total state employees in NC indicates that 41.6% of state employees, which includes LEOs, are located in urban counties, 20.4% in suburban counties, and 37.3% in rural counties [38].

### 2.2. Participants

The inclusion criteria of The Carolina Blue Project included individuals holding the official title of sworn police officer, police cadet, detention officer, or deputy, and any other LEOs actively engaged in their duties within the state of NC. Additionally, participants were required to be at least 18 years old. A total of 371 potential respondents were approached. Out of these, twenty-four were found to be participating in multiple surveys simultaneously, forty-eight initiated the survey but provided no responses, and sixteen participants were ineligible for inclusion in the study. Consequently, two hundred and eighty-three LEOs from over 40 NC counties participated in the project.

### 2.3. Procedure

All participants from The Carolina Blue Project gave their informed consent for inclusion before they participated in the project. The project was conducted in accordance with the Declaration of Helsinki, and the protocol was approved by the Ethics Committee of the University of North Carolina at Chapel Hill (approval no. 22-2052). The data collection took place from 1 February to 30 June 2023. The Carolina Blue Project research team employed three methods to recruit participants. Initially, the team collaborated extensively with administrators from various law enforcement agencies in NC to effectively communicate the study’s objectives and actively encourage their involvement through presentations, flyers, and brochures. Furthermore, participant recruitment occurred through “The Carolina Blue Project” website, which provided a comprehensive project summary and facilitated access to the REDCap survey link. Additionally, social media platforms, such as Facebook and Twitter, were utilized to engage participants in the study. These platforms were used to disseminate recruitment information and share the project website link. Participants who chose to participate had the option to access the REDCap survey electronically via the project website and social media platforms. Moreover, the survey could be accessed through a QR code provided on the project’s flyers and brochures. Participants were required to electronically complete the consent form, verify their eligibility by responding to screening inquiries related to their age and occupation, and then proceed to complete the survey.

### 2.4. Measures

#### 2.4.1. Demographic and Work-Related Variables

Participants were asked to report their age, sex, education, race, job titles, whether they served in leadership positions (yes/no), the NC county they were currently working in, and whether they had required rotation shifts (yes/no), required patrol duties (yes/no), and experience of being exposed to any toxic materials at work, such as (but not limited to) cocaine, heroin, or fentanyl (yes/no). We classified job categories according to the job titles as follows: (1) Police Officer, including Police Officer, Inspector, Investigator, Sergeant, Detective, Captain, Lieutenant, K9 handler, Major, Park Ranger, Wildlife Officer, Public Safety Officer, School Officer, Police Cadet, and Community Resource Officer; (2) Deputy Sheriff and Trooper; and (3) Other, including Probation and Parole Officer, Correctional Officer, Detention Officer, and Transportation Officer. In addition, the participants’ self-reported work county was classified as urban county, suburban county, or rural county.

#### 2.4.2. Burnout

Burnout was assessed using the Maslach Burnout Inventory (MBI), which consists of a total of 22 items [39]. The MBI encompasses three subscales: emotional exhaustion (MBI-EE; nine items), depersonalization (MBI-DEP; five items), and personal accomplishment (MBI-PA; eight items) [39]. MBI-EE measures the extent of emotional burden and fatigue experienced in relation to work, while MBI-DEP measures the increased mental distance from one’s job, feelings of negativism or cynicism related to one’s job, or individuals’ levels of indifference and impersonal attitudes toward others [40]. MBI-PA evaluates self-efficacy and feelings of accomplishment at work [40]. Each item employs a 7-point scale ranging from 0 to 6 (0 = never; 1 = at least a few times a year; 2 = at least once a month; 3 = several times a month; 4 = once a week; 5 = several times a week; and 6 = every day) [40]. Higher sum scores on the MBI subscales indicate higher levels of emotional exhaustion, depersonalization, or personal accomplishment [39]. Previous research reported Cronbach’s alpha coefficients in the range from 0.84 to 0.90 for MBI-EE, 0.74 to 0.84 for MBI-DEP, and 0.70 to 0.78 for MBI-PA [41]. The Cronbach’s alpha was 0.77 for MBI-EE, 0.90 for MBI-DEP, and 0.65 for MBI-PA in the current study.

#### 2.4.3. Operational Police Stress

Operational police stress was assessed using the Operational and Organizational Police Stress Questionnaires (PSQs) [42]. The PSQ comprises two distinct subscales: the Operational Police Stress Questionnaire (PSQ-Op) and the Organizational Police Stress Questionnaire (PSQ-Org) [42]. The PSQ-Op is designed to evaluate stressors related to job performance, while the PSQ-Org assesses stressors associated with the organization and culture in which individuals work [42]. Our study specifically employed the PSQ-Op to measure operational police stress, aligning with our objective to gain a nuanced understanding of stressors directly linked to job performance among LEOs. The PSQ-Op consists of 20 items, and responses are assessed on a 7-point scale ranging from 1, indicating no stress at all, to 7, indicating a lot of stress [42]. A higher sum score on the PSQ-Op indicates higher levels of operational police stress [42]. A prior study reported a Cronbach’s alpha of 0.93 for the PSQ-Op [43]. The Cronbach’s alpha for the PSQ-Op was 0.94 in the current study.

#### 2.4.4. Depression, Anxiety, and Stress

Depression, anxiety, and stress were assessed using the Depression, Anxiety, and Stress Scale (DASS-21) [44]. The DASS-21 is a 21-item questionnaire with three subscales: DASS-depression (7 items), DASS-anxiety (7 items), and DASS-stress (7 items), which measure the severity of these symptoms over the preceding week using a 4-point scale (0 = did not apply at all; 1 = applied to me to some degree, or some of the time; 2 = applied to me a considerable degree, or a good part of the time; 3 = applied to me very much, or most of the time [44]. Higher sum scores on the DASS-21 subscales indicate higher levels of depression, anxiety, or stress [44]. Zanon et al. [45] reported values of Cronbach’s alpha for the depression, anxiety, and stress subscales as 0.87, 0.83, and 0.87, respectively. Cronbach’s alpha for the depression, anxiety, and stress subscales in the current study were 0.88, 0.73, and 0.81, respectively.

#### 2.4.5. PTSD Symptoms

PTSD symptoms were measured by the Post-traumatic Stress Disorder Checklist (PCL-5), a self-reported measure comprising 20 items that assesses the symptoms of PTSD outlined in the Diagnostic and Statistical Manual of Mental Disorders, Fifth Edition [46]. The rating scale for each symptom ranges from 0 to 4 (0 = not at all; 1 = a little bit; 2 = moderately; 3 = quite a bit; and 4 = extremely) [46]. A higher sum score on the PLC-5 indicates a greater severity of PTSD symptoms [46]. The Cronbach’s alpha was 0.94 in a previous study [46] and 0.95 in the current study.

### 2.5. Statistical Analyses

Descriptive statistics (mean and standard deviation, or frequency and percentage, as appropriate) were computed for demographic and work-related variables, as well as for the scores of all the questionaries. Comparative statistics were performed using independent sample *t*-tests, one-way ANOVA, Mann–Whitney U tests, and Kruskal–Wallis tests. The sum scores of MBI-EE, MBI-DEP, MBI-PA, PSQ-Op, DASS-depression, DASS-anxiety, DASS-stress, and PCL-5 served as outcome variables. We examined histograms of these outcome variables to assess normality. Independent *t*-tests and one-way ANOVA were utilized to compare the means of normally distributed outcome variables (i.e., MBI-EE, MBI-DEP, MBI-PA, and DASS-stress) from among the demographic and work-related variables (i.e., sex, education, race, job category, service in leadership positions, county type, required rotation shifts, required patrol duties, and exposure to toxic materials at work). Mann–Whitney U and Kruskal–Wallis tests were applied to compare the means of non-normally distributed outcome variables (i.e., PSQ-Op, PCL-5, DASS-depression, and DASS-anxiety) from among the above demographic and work-related variables. The percentage of missing data ranges from 0.3% to 22.61% across all the variables. All outcome variables have more than 5% missing. All missing data were input using the Expectation–Maximization method before conducting the comparative statistics analyses [47]. Two-sided *p*-values of 0.05 or less were considered statistically significant. All data were analyzed using the SAS 9.4 software [48]. Two-sided *p*-values of less than 0.05 were considered statistically significant.

## 3. Results

### 3.1. Participant Characteristics

Table 1 shows the characteristics of the participants. The study comprised a total of 283 participants, with an average age of 37.23 ± 8.75 years. The majority of the participants were male (67.14%), Caucasian (78.06%), with a baccalaureate degree (48.04%), did not serve in a leadership position (71.89%), were currently working in a suburban (36.79%) or rural county (36.79%), and had experienced exposure to some form of toxic material at work (60.42%).

### 3.2. Differences in Burnout between/among Groups

Table 2 presents the results of comparing burnout based on the LEOs’ demographic and work-related variables. Male participants demonstrated higher mean depersonalization scores than their female counterparts. Scheffe’s post hoc test revealed that LEOs working in rural counties exhibited significantly higher depersonalization mean values compared with those in urban counties (*p* < 0.01). Additionally, LEOs in rural (*p* < 0.01) and suburban (*p* < 0.01) counties demonstrated significantly higher mean values of personal accomplishment than their urban counterparts. Participants exposed to toxic materials at work displayed higher mean levels of emotional exhaustion (*p* = 0.03) and depersonalization (*p* < 0.01) than those without such exposure.

### 3.3. Differences in Operational Police Stress between/among Groups

Table 3 presents the results of comparing operational police stress scores based on the LEOs’ demographic and work-related variables. Participants engaged in patrol duties exhibited a higher mean level of operational police stress than those who did not (*p* = 0.03). Additionally, participants exposed to toxic materials at work displayed a higher mean level of operational police stress than those who had not been exposed (*p* < 0.01).

### 3.4. Differences in Depression, Anxiety, and Stress between/among Groups

Table 4 illustrates the results of comparing DASS-depression, anxiety, and stress scores based on the LEOs’ demographic and work-related variables. Significant differences were observed in the mean scores for depression, anxiety, and stress among different race groups and county types. Post hoc tests revealed that Caucasian officers had higher mean depression (*p* < 0.01), anxiety (*p* < 0.01), and stress scores (*p* < 0.01) compared with African American officers. In addition, LEOs who worked in rural counties had higher mean scores of depression, anxiety, and stress compared with LEOs who worked in urban (*p* < 0.01) or suburban (*p* = 0.01) counties. LEOs exposed to toxic materials at work exhibited higher mean levels of depression (*p* < 0.01), anxiety (*p* = 0.01), and stress (*p* < 0.01) than those not exposed to such materials at work.

### 3.5. Differences in Depression, Anxiety, and Stress between/among Groups

Table 5 presents the results of comparing PCL-5 scores based on the LEOs’ demographic and work-related variables. Significant differences were identified in the mean scores of PCL-5 among the three county types. LEOs who worked in rural counties had a higher mean score of PCL-5 compared with those who worked in urban counties (*p* < 0.01). Participants exposed to toxic materials at work exhibited a higher mean score of PCL-5 than those who had not been exposed (*p* = 0.01).

## 4. Discussion

The primary objective of the research was to investigate the severity of work-related stress and psychological distress among LEOs in NC, considering factors such as race, geography, and exposure to toxic materials at work. Overall, our findings indicate significant differences in the severity of work-related stress and psychological distress among different county types, exposure to toxic materials at work, and race/sex groups. Our findings show that LEOs working in rural areas reported higher levels of burnout related to depersonalization and personal achievement and higher mean scores for depression, anxiety, stress, and PTSD symptoms compared with their urban counterparts. Our finding is consistent with a previous study that found that a high level of burnout in 56.1% of paramedics, police, community nurses, and child protection workers in rural areas [49]. Our finding is also consistent with previous studies showing that rural officers, who are faced with longer backup times, isolation, and tasked with policing larger areas, exhibit higher stress levels than their urban counterparts [15,50]. Literature suggests that top stressors among LEOs working in rural areas could be staff shortages, bureaucratic challenges, and inconsistent leadership in rural policing contexts [50,51,52]. In addition, the overwhelming majority of NC counties remain rural in nature [36]. North Carolina has 100 counties, with 78 of them being rural and covering 71% of the state’s land [36]. The rate of LEOs per 1000 NC residents is 1.81 in NC rural counties and 2.26 in NC urban counties [53]. Although the majority of NC’s residents (57%; 5.9 million people) live in urban counties, 4.6 million people (43%) still reside in rural counties [54]. Given that the majority of land in the state of NC is rural, a significant portion of the population resides in rural counties, and there is a lower number of LEOs per 1000 residents in rural counties compared with urban counties, it is no surprise that rural LEOs carry an overwhelming burden in their line of work, which may contribute to the adverse mental health outcomes we observed in our study. Furthermore, although our study found no statistically significant difference in the levels of burnout, operational police stress, depression, anxiety, stress, and PTSD among the three job categories in our sample (i.e., 1. Police officers; 2. Deputy sheriffs and troopers; and 3. Probation and parole officers, correctional officers, and detention officers), it is important to note that these job categories have different jurisdictions in North Carolina. Police officers serve within the boundaries of the city or municipality they work in. Deputy sheriffs work for county sheriff’s offices and cover the entire county. Troopers work for the NC State Highway Patrol and focus on traffic enforcement and highway safety on state highways and interstate roadways throughout the state. Probation and parole officers’ jurisdiction may include specific regions within the state of NC. Correctional officers and detention officers work in state prisons and county jails, respectively [55]. Future studies should further explore the unique factors that influence the mental health of LEOs in NC’s rural counties by qualitative research methods such as in-depth interviews, focus groups, and participant observations to provide valuable insights into the challenges these officers face in their specific rural areas.

Our findings indicate that LEOs who reported exposure to toxic materials at work demonstrated higher mean scores for burnout related to emotional exhaustion and depersonalization, operational police stress, depression, anxiety, stress, and PTSD symptoms compared with their counterparts who did not report such exposure. The heightened impact of toxic material exposure on LEOs’ well-being aligns with the existing literature highlighting the diverse chemical risks they face [56,57]. LEOs are frequently exposed to toxic materials, presenting chemical risks that include contact with drugs (e.g., methylamphetamine, fentanyl), toxic gases resulting from combustion processes during fires (e.g., polycyclic aromatic hydrocarbons, dioxins, furans, formaldehyde), substances released in the event of chemical accidents (e.g., solvents, pesticides), self-defense sprays, and a diverse array of materials and reagents used in forensic laboratories [56,57]. Consistent with the findings of our study, other research studies also support the link between toxic material exposure and work-related stress and psychological distress [27,58]. For instance, in a Dutch study involving a cohort of 1468 LEOs, of which 834 were exposed and 634 were non-exposed to toxic materials, those who were exposed reported significantly more symptoms of anxiety, depression, somatic complaints, fatigue, sleep disturbances, and PTSD compared with their non-exposed counterparts [58]. While previous studies have mainly concentrated on the physical health effects of toxic material exposure on LEOs, such as cancer or lung disease [59,60], our research has uncovered new evidence indicating that exposure to toxic materials in the workplace can also have a significant impact on the mental health of LEOs. Public-health experts, healthcare providers, and healthcare researchers should work in collaboration with leaders of law enforcement agencies to provide a comprehensive healthcare approach that includes coping mechanisms and mental health support services. This will help ensure the well-being and resilience of LEOs who are exposed to toxic materials in the course of their work.

Our findings revealed that Caucasian LEOs had higher mean scores for depression, anxiety, and stress compared with their African American counterparts, and male LEOs had higher mean scores of burnout related to depression than female LEOs. Previous studies on race-based mental health in LEOs showed mixed results [17,61,62]. Our finding is consistent with a previous study in which Caucasian LEOs reported statistical significantly higher levels of depression, anxiety, and stress compared with African American LEOs across nine Baltimore cities in the U.S. [17]. However, another study found that LEOs of color experienced higher levels of police organizational stress than Caucasian LEOs [62]. Law enforcement in the U.S. has historically been dominated by Caucasian men, which may lead to the general assumption that minority LEOs, especially African American or female officers, face greater mental health challenges than their Caucasian counterparts due to prejudice, a lack of mentors and role models, and a lack of support from supervisors and colleagues [61,63]. In NC, the racial distribution of LEOs as follows: 74% Caucasian, 15% African American, 4% Hispanic, and 2% Asian [64]. However, our findings appear to contradict this commonly held assumption. Several plausible explanations account for our observed findings. First, our study was conducted three years after the tragedy in which Mr. George Floyd, an African American citizen, was murdered by mainly Caucasian LEOs during a police arrest in 2020 [65]. Thus, Caucasian LEOs in NC may experience higher work-related stress when performing their duties due to the anti-police climate in recent years [66]. Second, although we used non-parametric tests (i.e., the Kruskal–Wallis test for race groups and the Mann–Whitney U test for sex groups) for comparative statistics due to the non-normally distribution of our outcome variables, our sample was mostly composed of Caucasian males, which might have affected our results. Third, only 13% of LEOs in NC are female [64]. The literature has shown that in the law enforcement field, which is predominantly occupied by males, female officers are often given administrative duties that are considered less valuable [67]. This may be a contributing factor to the difference in burnout levels observed between the male and female LEOs in our study. Given the complex interplay of race, gender, and recent sociopolitical events in shaping the mental health outcomes of LEOs, future research should investigate the mental well-being of LEOs based on different race and gender groups and explore the potential occupational disparities faced by female LEOs in predominantly male-dominated law enforcement environments to provide deeper insights into the factors influencing mental health outcomes in diverse policing contexts, which would ultimately inform targeted interventions and support systems.

This study has several limitations. First, the definition of toxic materials is not clearly defined in our questionnaires, and the assessment of exposure to toxic materials at work was limited to a binary response (yes/no), which may not fully capture the impact of exposure to different toxic materials on work-related stress and psychological distress. To the best of our knowledge, no hazardous material survey is specifically designed for law enforcement officers. Future studies should consider refining the definition and measurement of exposure to toxic/hazardous materials or harmful substances, particularly in the context of law enforcement work, to better understand its impact on work-related stress and psychological distress. Second, as this is an exploratory study, we did not perform a power analysis to determine the necessary sample size for our statistical tests before recruiting participants. This could potentially limit our statistical power to detect significant differences in our outcome variables across various groups. Third, in our analysis, we did not consider the number of years of service as an LEO or include age in our comparative statistical approaches. This lack of information may limit our understanding of how age or the number of years of service as an LEO can affect the levels of burnout, operational police stress, depression, anxiety, stress, and PTSD in our sample. Fourth, the findings cannot be assumed to be generalizable to the population of NC law enforcement since most participants were Caucasian male LEOs. Fifth, the cross-sectional design limits the ability to establish causal relationships and generalize findings. In addition, due to the voluntary participation nature of our method, our sample proportions in the urban, suburban, and rural counties do not fully match the overall state employee population proportions in NC, which may limit the generalizability of our findings. Finally, our findings cannot indicate clinical levels of depression and anxiety because the DASS-21 is not a clinical diagnostic tool and can only suggest the severity of depression and anxiety symptoms based on self-reported data [45].

## 5. Conclusions

Our research focuses on work-related stress and psychological distress among LEOs in NC. The findings indicate that LEOs working in NC rural areas, those who are exposed to toxic materials, and Caucasian male LEOs experience higher levels of work-related stress and psychological distress in comparison with their counterparts. Our findings could impact not only LEOs in NC but also other first-responders nationwide. It is crucial for leaders in law enforcement departments to be aware of the severity of work-related stress and psychological distress among LEOs and to take necessary measures to prioritize their overall well-being and psychological health. By implementing supportive systems, training programs, and fostering adaptive work environments, the impact of work-related stressors can be mitigated, and psychological distress on LEOs can be reduced.

## Figures and Tables

**Table 1:** Sample characteristics (total n = 283).

Characteristics	Mean (SD) or n (%)	
Age	37.23	(8.75)
Sex		
Male	189	(67.14%)
Female	92	(32.86%)
Education		
Less than baccalaureate degree	105	(37.37%)
Baccalaureate degree	135	(48.04%)
Graduate	41	(14.59%)
Race		
Caucasian	217	(78.06%)
African American	43	(15.47%)
Other ^†^	18	(6.47%)
Job category		
Police Officer	181	(64.41%)
Deputy Sheriff, Trooper	29	(10.32%)
Probation and Parole Officer, Correctional Officer, Detention Officer	71	(25.27%)
Leader		
Yes	79	(28.11%)
No	202	(71.89%)
County type		
Urban county	74	(26.42%)
Suburban county	103	(36.79%)
Rural county	103	(36.79%)
Required to rotate shifts		
Yes	56	(19.79%)
No	227	(80.21%)
Required to perform patrol duties		
Yes	170	(60.07%)
No	113	(39.93%)
Exposed to any toxic materials at work		
Yes	171	(60.42%)
No	112	(39.58%)

*Note*: ^†^ = American Indian/Native Alaskan, Asian, or mixed race.

**Table 2:** Comparing burnout scores by police officers’ demographics and work-related variables.

Characteristics	MBI-EE			MBI-DEP			MBI-PA		
	N	Mean (SD)	Test and *p*-Value	N	Mean (SD)	Test and *p*-Value	N	Mean (SD)	Test and *p*-Value
Sex									
Male	189	22.56 (10.56)	*t* = −1.62 *p* = 0.11	189	10.40 (5.39)	*t* = 2.04 *p* = 0.04	189	33.53 (8.05)	*t* = 1.94 *p* = 0.05
2. Female	92	24.74 (10.79)		92	8.98 (5.66)		92	31.43 (9.45)	
Education									
Less than baccalaureate degree	105	22.00 (9.77)	*F* = 2.63 *p* = 0.07	105	9.80 (5.46)	*F* = 1.96 *p* = 0.14	105	33.97 (8.63)	*F* = 2.66 *p* = 0.07
2. Baccalaureate degree	135	24.85 (11.69)		135	10.38 (5.49)		135	32.33 (8.52)	
3. Graduate degree	41	21.41 (9.73)		41	8.45 (5.61)		41	30.41 (9.42)	
Race									
Caucasian	217	22.87 (10.58)	*F* = 1.45 *p* = 0.24	217	10.20 (5.52)	*F* = 2.33 *p* = 0.10	217	33.10 (8.35)	*F* = 1.31 *p* = 0.27
2. African American	43	25.60 (11.92)		43	8.37 (5.41)		43	30.84 (10.29)	
3. Other ^†^	18	21.31 (9.66)		18	8.86 (4.79)		18	31.66 (9.50)	
Job Category									
Police Officer	181	22.60 (10.76)	*F* = 0.93 *p* = 0.40	181	10.29 (5.51)	*F* = 1.41 *p* = 0.25	181	29.72 (7.12)	*F* = 2.47 *p* = 0.09
2. Deputy Sheriff, Trooper	29	23.71 (9.67)		29	9.78 (5.61)		29	31.48 (6.83)	
3. Probation and Parole Officer, Correctional Officer, Detention Officer	71	24.62 (11.62)		71	9.00 (5.45)		71	28.02 (8.54)	
Served in a leadership position									
Yes	79	23.85 (10.52)	*t* = 0.61 *p* = 0.54	79	10.59 (5.66)	*t* = 1.28 *p* =0.20	79	32.50 (8.30)	*t* = −0.22 *p* = 0.83
2. No	202	22.98 (10.88)		202	9.65 (5.45)		202	32.76 (8.93)	
County type									
Urban	74	22.28 (12.34)	*F* = 1.09 *p* = 0.34	74	8.29 (5.21)	*F* = 5.41 *p* = 0.0049 post hoc: 3 > 1 **	74	29.37 (10.44)	F = 8.10 *p* = 0.0004 post hoc: 2 > 1 ** 3 > 1 **
2. Suburban	102	22.65 (10.86)		102	9.77 (5.49)		102	34.53 (7.99)	
3. Rural	103	24.45 (9.50)		103	11.01 (5.48)		103	33.15 (7.48)	
Required to rotate shifts									
Yes	56	24.23 (8.83)	*t* = −0.81 *p* = 0.42	56	9.35 (5.22)	*t* = 0.88 *p* = 0.38	56	29.98 (7.71)	*t* = −0.59 *p* = 0.56
2. No	225	23.11 (11.11)		225	10.08 (5.56)		225	29.49 (7.22)	
Required to perform patrol duties									
Yes	168	23.45 (10.72)	*t* = −0.22 *p* = 0.83	168	9.62 (5.40)	*t* = 1.11 *p* = 0.27	168	30.01 (7.27)	*t* = −1.16 *p* = 0.25
2. No	113	23.15 (10.70)		113	10.36 (5.60)		113	29.02 (7.31)	
Exposure to toxic materials									
Yes	169	24.41 (10.38)	*t* = −2.12 *p* = 0.03	169	11.00 (5.53)	*t* = −3.96 *p* < 0.0001	169	32.55 (7.92)	*t* = 1.23 *p* = 0.22
2. No	112	21.68 (10.74)		112	8.43 (5.01)		112	33.79 (8.81)	

*Note*: MBI-EE = Maslach Burnout Inventory—Emotional exhaustion; MBI-DEP = Maslach Burnout Inventory—Depersonalization; MBI-PA = Maslach Burnout Inventory—Personal accomplishment; t = independent *t*-Test; F = one-way ANOVA; ^†^ = American Indian/Native Alaskan, Asian, or mixed race; ** = *p* < 0.01.

**Table 3:** Comparing operational police stress scores by police officers’ demographics and work-related variables.

Characteristics	N	Mean (SD)	Test and *p*-Value
Sex			
Male	189	3.22 (1.19)	*U* = 13,053.50 *p* = 0.90
2. Female	92	3.25 (1.14)	
Education			
Less than baccalaureate degree	105	3.14 (1.17)	*X*^2^ = 4.07 *p* = 0.13
2. Baccalaureate degree	135	3.38 (1.16)	
3. Graduate degree	41	3.01 (1.28)	
Race			
Caucasian	217	3.22 (1.20)	*X*^2^ = 0.13 *p* = 0.94
2. African American	43	3.27 (1.10)	
3. Other ^†^	18	3.18 (1.25)	
Job Category			
Police Officer	181	3.29 (1.17)	*X*^2^ = 2.34 *p* = 0.31
2. Deputy Sheriff, Trooper	29	3.08 (1.23)	
3. Probation and Parole Officer, Correctional Officer, Detention Officer	71	3.13 (1.19)	
Served in a leadership position			
Yes	79	3.40 (1.10)	*U* = 12,228.00 *p* = 0.08
2. No	202	3.16 (1.21)	
County type			
Urban	74	3.03 (1.08)	*X*^2^ = 4.18 *p* = 0.12
2. Suburban	102	3.23 (1.30)	
3. Rural	103	3.38 (1.13)	
Required to rotate shifts			
Yes	56	3.47 (1.27)	*U* = 8795.50 *p* = 0.10
2. No	225	3.18 (1.15)	
Required to perform patrol duties			
Yes	168	3.34 (1.16)	*U* = 14,464.00 *p* = 0.03
2. No	113	3.07 (1.18)	
Exposure to toxic materials			
Yes	169	3.42 (1.18)	*U* = 13,533.00 *p* = 0.0007
2. No	112	2.95 (1.09)	

*Note*: U = The Mann–Whitney U test; *X*^2^ = Kruskal–Wallis test; ^†^ = indicates American Indian/Native Alaskan, Asian, or mixed race.

**Table 4:** Comparing depression, anxiety, and stress scores by police officers’ demographics and work-related variables.

Characteristics	DASS-Depression			DASS-Anxiety			DASS-Stress		
	N	Mean (SD)	Test and *p*-Value	N	Mean (SD)	Test and *p*-Value	N	Mean (SD)	Test and *p*-Value
Sex									
Male	189	6.95 (6.61)	*U* = 11,798.50 *p* = 0.06	189	4.78 (4.65)	*U* = 12,330.00 *p* = 0.31	189	10.98 (6.63)	*t* = 0.55 *p* = 0.58
2. Female	92	5.51 (5.91)		92	4.17 (4.35)		92	10.53 (6.29)	
Education									
Less than baccalaureate degree	105	6.71 (5.66)	*X*^2^ = 3.74 *p* = 0.15	105	4.37 (4.08)	*X*^2^ = 2.79 *p* = 0.25	105	10.16 (6.30)	*F* = 2.73 *p* = 0.07
2. Baccalaureate degree	135	6.93 (7.49)		135	5.08 (5.24)		135	11.70 (7.09)	
3. Graduate degree	41	4.51 (3.99)		41	3.14 (2.85)		41	9.42 (4.80)	
Race									
Caucasian	217	7.08 (6.78)	*X*^2^ = 8.53 *p* = 0.0141 Post hoc: 1 > 2 **	217	4.87 (4.68)	*X*^2^ = 8.87 *p* = 0.0119 Post hoc: 1 > 2 **	217	11.48 (6.62)	*F* =7.17 *p* = 0.0009 Post hoc: 1 > 2 **
2. African American	43	4.05 (4.20)		43	2.67 (2.85)		43	8.08 (4.58)	
3. Other ^†^	18	4.86 (4.58)		18	4.51 (4.49)		18	7.94 (5.47)	
Job Category									
Police Officer	181	6.94 (6.39)	*X*^2^ = 5.53 *p* = 0.06	181	4.72 (4.66)	*X*^2^ = 2.24 *p* = 0.33	181	11.16 (6.34)	*F* = 0.93 *p* = 0.40
2. Deputy Sheriff, Trooper	29	5.63 (8.04)		29	4.37 (3.93)		29	9.57 (7.45)	
3. Probation and Parole Officer, Correctional Officer, Detention Officer	71	5.59 (5.88)		71	4.09 (4.61)		71	10.39 (6.68)	
Served in a leadership position									
Yes	79	7.33 (6.90)	*U* = 12,001.50 *p* = 0.16	79	4.56 (4.42)	*U* = 11,372.00 *p* = 0.70	79	11.62 (6.38)	*t* = 1.30 *p* = 0.19
2. No	202	6.13 (6.27)		202	4.51 (4.64)		202	10.49 (6.60)	
County									
Urban	74	4.36 (4.92)	*X*^2^ = 19.74 *p* = <0.0001 Post hoc: 3 > 1 ** 3 > 2	74	3.39 (3.82)	*X*^2^ = 10.24 *p* = 0.006 Post hoc: 3 > 1 **	74	8.11 (5.71)	*F* = 8.34 *p* = 0.0003 Post hoc: 3 > 1 ** 2 > 1
2. Suburban	102	6.04 (6.25)		102	4.45 (4.45)		102	10.92 (6.92)	
3. Rural	103	8.47 (7.10)		103	5.43 (4.94)		103	12.40 (6.22)	
Required to rotate shifts									
Yes	56	6.35 (5.51)	*U* = 8097.50 *p* = 0.71	56	5.00 (4.06)	*U* = 8803.50 *p* = 0.09	56	11.53 (5.80)	*F* = −0.94 *p* = 0.35
2. No	225	6.49 (6.69)		225	4.41 (4.69)		225	10.61 (6.71)	
Required to perform patrol duties									
Yes	168	6.77 (6.39)	*U* = 14,917.50 *p* = 0.09	168	4.69 (4.63)	*U* = 15,276.00 *p* = 0.32	168	10.97 (6.58)	*F* = −0.63 *p* = 0.53
2. No	113	5.98 (6.55)		113	4.27 (4.49)		113	10.46 (6.51)	
Exposure to toxic materials									
Yes	169	7.56 (6.85)	*U* = 13,499.50 *p* = 0.0005	169	4.84 (4.20)	*U* = 14,173.00 *p* = 0.0142	169	11.87 (6.52)	*t* = −3.39 *p* = 0.0008
2. No	112	4.82 (5.32)		112	4.13 (5.03)		112	9.22 (6.21)	

*Note*: DASS-Depression = Depression subscale of the Depression, Anxiety, and Stress Scale; DASS-Anxiety = Anxiety subscale of the Depression, Anxiety, and Stress Scale; DASS-Stress = Stress subscale of the Depression, Anxiety, and Stress Scale; t = independent *t*-Test; F = one-way ANOVA; U = Mann–Whitney U test; *X*^2^ = Kruskal–Wallis test; ^†^ = indicates American Indian/Native Alaskan, Asian, or mixed race; ** = *p* < 0.01.

**Table 5:** Comparing Post-traumatic Stress Disorder Checklist (PCL-5) scores by police officers’ demographics and work-related variables.

Characteristics	N	Mean (SD)	Test and *p*-Value
Sex			
Male	189	17.60 (14.51)	*U* = 13,496.00 *p* = 0.41
2. Female	92	18.39 (13.92)	
Education			
Less than baccalaureate degree	105	17.98 (13.22)	*X*^2^ = 0.44 *p* = 0.80
2. Baccalaureate degree	135	18.17 (15.89)	
3. Graduate degree	41	15.95 (11.31)	
Race			
Caucasian	217	18.05 (14.56)	*X*^2^ = 0.23 *p* = 0.89
2. African American	43	16.64 (13.87)	
3. Other ^†^	18	17.57 (12.85)	
Job Category			
Police Officer	181	17.73 (13.72)	*X*^2^ = 0.61 *p* = 0.74
2. Deputy Sheriff, Trooper	29	15.79 (12.79)	
3. Probation and Parole Officer, Correctional Officer, Detention Officer	71	18.72 (15.98)	
Served in a leadership position			
Yes	79	18.50 (14.21)	*U* = 11,641.50 *p* = 0.41
2. No	202	17.50 (14.23)	
County			
Urban	74	14.01 (13.10)	*X*^2^ = 12.49 *p* = 0.0019 Post hoc: 3 > 1 **
2. Suburban	102	17.34 (14.56)	
3. Rural	103	20.87 (13.80)	
Required to rotate shifts			
Yes	56	19.13 (13.72)	*U* = 8433.50 *p* = 0.32
2. No	225	17.63 (14.38)	
Required to perform patrol duties			
Yes	168	18.13 (13.44)	*U* = 15,214.50 *p* = 0.28
2. No	113	17.29 (15.34)	
Exposure to toxic materials			
Yes	169	19.50 (14.37)	*U* = 14,105.50 *p* = 0.0114
2. No	112	15.58 (13.76)	

*Note*: PTSD scale = Post-traumatic Stress Disorder Checklist for DSM-5; *U* = Mann–Whitney U test; *X*^2^ = Kruskal–Wallis test; ^†^ = indicates American Indian/Native Alaskan, Asian, or mixed race; ** = *p* < 0.01.

## Data Availability

Data are contained within the article.
